# Medications and addictive substances potentially inducing or attenuating sleep bruxism and/or awake bruxism

**DOI:** 10.1111/joor.13061

**Published:** 2020-08-10

**Authors:** Cees de Baat, Merel Verhoeff, Jari Ahlberg, Daniele Manfredini, Ephraim Winocur, Petra Zweers, Fred Rozema, Arjan Vissink, Frank Lobbezoo

**Affiliations:** ^1^ Foundation for Oral Health and Parkinson’s Disease Oegstgeest The Netherlands; ^2^ Fresh Unieke Mondzorg Woerden The Netherlands; ^3^ Department of Oral Function and Prosthetic Dentistry Radboud university medical center Nijmegen The Netherlands; ^4^ Department of Orofacial Pain and Dysfunction Academic Centre for Dentistry Amsterdam (ACTA) University of Amsterdam and Vrije Universiteit Amsterdam Amsterdam The Netherlands; ^5^ Department of Oral and Maxillofacial Diseases University of Helsinki Helsinki Finland; ^6^ Department of Biomechanical Diseases School of Dentistry University of Siena Siena Italy; ^7^ Department of Oral Rehabilitation Dental School Tel Aviv University Tel Aviv Israel; ^8^ Netherlands pharmacovigilance centre LAREB Hertogenbosch The Netherlands; ^9^ Department of Oral Medicine Academic Centre for Dentistry Amsterdam (ACTA) University of Amsterdam and Vrije Universiteit Amsterdam Amsterdam The Netherlands; ^10^ Department of Oral and Maxillofacial Surgery Amsterdam UMC University of Amsterdam Amsterdam The Netherlands; ^11^ Department of Oral and Maxillofacial Surgery University Medical Centre Groningen University of Groningen Groningen The Netherlands

**Keywords:** addictive, adverse effect, attenuating effect, awake bruxism, medication, sleep bruxism

## Abstract

Bruxism is a repetitive jaw‐muscle activity characterised by clenching or grinding of the teeth and/or by bracing or thrusting of the mandible. It can occur during sleep, indicated as sleep bruxism, or during wakefulness, indicated as awake bruxism. Exogenous risk indicators of sleep bruxism and/or awake bruxism are, among others, medications and addictive substances, whereas also several medications seem to have the potential to attenuate sleep bruxism and/or awake bruxism. The objective of this study was to present a narrative literature on medications and addictive substances potentially inducing or aggravating sleep bruxism and/or awake bruxism and on medications potentially attenuating sleep bruxism and/or awake bruxism. Literature reviews reporting evidence or indications for sleep bruxism and/or awake bruxism as an adverse effect of several (classes of) medications as well as some addictive substances and literature reviews on medications potentially attenuating sleep bruxism and/or awake bruxism were used as starting point and guidelines to describe the topics mentioned. Additionally, two literature searches were established on PubMed. Three types of bruxism were distinguished: sleep bruxism, awake bruxism and non‐specified bruxism. Generally, there are insufficient evidence‐based data to draw definite conclusions concerning medications and addictive substances inducing or aggravating sleep bruxism and/or awake bruxism as well as concerning medications attenuating sleep bruxism and/or awake bruxism. There are insufficient evidence‐based data to draw definite conclusions concerning medications and addictive substances inducing or aggravating sleep bruxism and/or awake bruxism as well as concerning medications attenuating sleep bruxism and/or awake bruxism.

## 1 INTRODUCTION

Bruxism is a repetitive jaw‐muscle activity characterised by clenching or grinding of the teeth and/or by bracing or thrusting of the mandible. Bruxism has two distinct circadian manifestations: it can occur during sleep, indicated as sleep bruxism, or during wakefulness, indicated as awake bruxism.^1^ In March 2017, an international consensus meeting, Assessment of Bruxism Status, with bruxism experts from around the globe developed separate definitions for sleep bruxism and awake bruxism. The new definitions are as follows. Sleep bruxism is a masticatory muscle activity during sleep that is characterised as rhythmic (phasic) or non‐rhythmic (tonic) and is not a movement disorder or a sleep disorder in otherwise healthy individuals. Awake bruxism is a masticatory muscle activity during wakefulness that is characterised by repetitive or sustained tooth contact and/or by bracing or thrusting of the mandible and is not a movement disorder in otherwise healthy individuals.^2^ Aetiologically, emerging evidence suggests that biologic, psychologic and exogenous risk indicators have greater involvement than morphologic factors.^3^ This narrative literature review zooms in on two exogenous risk indicators of bruxism: medications and addictive substances.

During the current century, literature reviews have been published reporting evidence or indications for bruxism as an adverse effect of several (classes of) medications as well as some addictive substances.^4^, ^5^, ^6^, ^7^ In this article, these literature reviews are used as starting point and guidelines to describe three topics: classes of medications, individual medications and addictive substances potentially inducing or aggravating sleep bruxism and/or awake bruxism. Additionally, a literature search was established on PubMed using terms related to “bruxism”, “adverse effect”, “medication”, and “addictive”. The focus for inclusion was a report or several reports on bruxism as adverse effect of a medication or an addictive substance, without any limitation or quality assessment. Reference lists of primary articles were also searched, and relevant articles not retrieved in the primary search were added. Furthermore, as fourth topic, a narrative review is presented on medications potentially attenuating sleep bruxism and/or awake bruxism. Five literature reviews were used as starting point and guidelines.^4^, ^5^, ^8^, ^9^, ^10^ Additionally, a similar literature search was established on PubMed using terms related to “bruxism”, “medication”, and “attenuating effect”. For each of the four topics, the medications and addictive substances are listed in subparagraphs in alphabetical order. When possible, each subparagraph is accomplished by a conclusion or a suggestion. In case a concluding remark is lacking, the information provided in the subparagraph is very weak, not justifying any conclusion or suggestion. Three types of bruxism are distinguished: sleep bruxism, awake bruxism and non‐specified bruxism.

## 2 CLASSES OF MEDICATIONS POTENTIALLY INDUCING BRUXISM

### 2.1 Anticonvulsants

In Brazil, a research project was carried out among children and adolescents with cerebral palsy, who did or did not receive anticonvulsants as medication, and a control group. According to their caregivers, statistically significantly more children and adolescents with cerebral palsy showed non‐specified bruxism than children and adolescents of the control group. Among the children and adolescents with cerebral palsy who received anticonvulsants, those who received barbiturates showed a greater frequency of non‐specified bruxism when compared to those who received other anticonvulsants.^11^

### 2.2 Phenethylamines

In a pilot study among adolescents suffering from attention deficit hyperactivity disorder (ADHD), a strong association was suggested between the medication class of phenethylamines (amphetamines and methylphenidate) and the number of teeth with attrition/wear facets, potentially due to sleep and/or awake bruxism.^12^ When compared to a control group, 281 adolescents diagnosed with ADHD were more likely to have current and lifetime sleep disorders, including bruxism. The use of methylphenidate was associated with a further increase of sleep bruxism.^13^ A group of 156 adults aged 18‐55 years and diagnosed with ADHD received extended‐release SHP465 mixed amphetamine salts, which are comprised of equal parts of dextroamphetamine sulphate, amphetamine sulphate, dextroamphetamine saccharate and amphetamine aspartate monohydrate. This medication reduced their ADHD symptoms statistically significantly when compared to a control group of 80 patients who received a placebo. However, five per cent of the adults who received SHP465 mixed amphetamine salts reported treatment‐emergent adverse events, including non‐specified bruxism.^14^

Besides these studies among groups of ADHD patients, three case reports have been published. A 9‐year‐old boy diagnosed with ADHD was prescribed 10 mg/day methylphenidate for fourteen days and 15 mg/day thereafter. During the fourth week of treatment, his parents noticed teeth grinding sounds when he was sleeping. Methylphenidate was discontinued after a week of sleep bruxism. Complete remission from bruxism was noticed immediately. Due to ADHD symptoms three weeks later, methylphenidate on the lower dose was reinitiated. Sleep bruxism recommenced ten days later. A wait‐and‐watch policy was adopted, whilst the boy continued to use 10 mg/day methylphenidate. The sleep bruxism spontaneously remitted less than two weeks after it began. There was no relapse during follow‐up for 10 weeks.^15^ The second case report presented a 9‐year‐old boy with ADHD who experienced severe awake bruxism after his second dose of sustained release of 18 mg/day methylphenidate. His mother discontinued the medication, and the bruxism remitted immediately. The bruxism was confirmed on rechallenge.^16^ Thirdly, a 9‐year‐old girl with ADHD received 18 mg/day methylphenidate. On day five, her parents reported audible teeth grinding during sleep, which dissipated totally with medication holidays on weekends.^17^

In conclusion: In ADHD patients treated with a high dose of phenethylamines, sleep bruxism and awake bruxism may present as an adverse effect.

### 2.3 Selective serotonin reuptake inhibitors

At the beginning of the current century, non‐specified bruxism was suggested to be an adverse effect of medication with selective serotonin reuptake inhibitors (SSRIs), following a questionnaire study among family physicians in the greater Amsterdam region.^18^ Recently, a systematic review of case reports on non‐specified bruxism as an adverse effect of SSRIs was published. Fluoxetine and sertraline were the most commonly reported offending medications.^19^ Children affected by ADHD taking SSRIs as medication showed a higher number of teeth with attrition/wear facets, potentially due to sleep and/or awake bruxism, when compared to control subjects as well as subjects affected by ADHD not taking medications. However, the association between taking phenethylamines and attrition/wear facets was more pronounced than the association between taking SSRIs and attrition/wear facets.^12^ A 6‐year‐old girl was diagnosed with separation anxiety disorder and received fluoxetine treatment. Subsequently, she presented intense sleep bruxism.^20^

It has been proposed that the pathophysiology of SSRI‐associated sleep and awake bruxism is the close relation between serotonin and dopamine in regulating motor pathways. An excess of serotonin in the nerve synapses may lead to an inhibitory effect on the release of dopamine, which plays a major role in movement control. However, scientific evidence for this theory of SSRI‐induced sleep and awake bruxism is still lacking.^4^, ^5^ An onset to scientific evidence has emerged from a systematic literature review on the association of the use of psychotropic medications with the presence of sleep bruxism. It was demonstrated that in adults, only the SSRI paroxetine was related to sleep bruxism with odds ratio 3.63 and 95% confidence interval 2.15‐6.13.^7^

In conclusion: Medication with SSRIs may induce sleep bruxism and/or awake bruxism.

## 3 INDIVIDUAL MEDICATIONS POTENTIALLY INDUCING BRUXISM

### 3.1 Aripiprazole

The atypical antipsychotic medication aripiprazole is used in the treatment of schizophrenia and bipolar disorder. The scientific literature reports only one case report on medication with aripiprazole and non‐specified bruxism as adverse effect.^21^ Therefore, it seems not very likely that bruxism is a frequently presenting adverse effect of aripiprazole.

### 3.2 Atomoxetine

Atomoxetine is a norepinephrine reuptake inhibitor used to reduce the symptoms of ADHD and as antidepressant. A systematic literature review of case reports on the use of antidepressants and bruxism as adverse effect revealed that sleep bruxism due to atomoxetine was mentioned in two out of 45 case reports.^19^ Not mentioned in this systematic review of case reports is an article on a 7‐year‐old boy with ADHD, who showed a clear onset and resolution of sleep bruxism as well as awake bruxism with the introduction and subsequent partial removal of atomoxetine. Furthermore, the sleep and awake bruxism symptoms worsened with higher dosages of atomoxetine.^22^

### 3.3 Duloxetine

Duloxetine is a serotonin‐norepinephrine reuptake inhibitor (SNRI) used to reduce symptoms of major depressive disorder, generalised anxiety disorder, diabetic neuropathy, fibromyalgia and chronic pain. A systematic literature review on the association of the use of psychotropic medications with the presence of sleep bruxism mentioned that duloxetine was positively related to sleep bruxism with odds ratio 2.16 and 95% confidence interval 1.12‐4.17.^7^

### 3.4 Flecainide

Flecainide is a medicament for treatment of ventricular and supraventricular tachycardias and dangerous cardial arrhythmias. In the scientific literature, only one case report has been published. It was on a 55‐year‐old man who developed awake bruxism and other involuntary oro‐facial movements within three days of initiation of flecainide.^23^ The fact that since 1992 no other case reports have been published, does suggest that bruxism is not a frequent adverse effect of flecainide.

### 3.5 Ketotifen

A 4‐year‐old boy received orally for one month 5 mg/day of the selective antihistamine ketotifen due to bronchospasm and rhinitis. Additionally, the boy was treated with beclomethasone by aerosol inhalation for one week. Ten days after beginning the treatment, the boy's parents noticed nightly episodes of bruxism. The sleep bruxism abruptly disappeared after withdrawal of ketotifen. Autonomously, ketotifen was readministered for a subsequent episode of bronchospasm, one month later. Sleep bruxism reappeared, exclusively in concomitance with the period of treatment. During one year of follow‐up, the boy did not experience any other bruxism episodes.^24^ The adverse effect of bruxism due to ketotifen was reasonably convincing, but additional reports are lacking.

### 3.6 Methadone

Methadone is an opioid used in case of other opioid dependence or chronic pain. Indications in case of reduction or cessation of other opioid use are maintenance or detoxification to manage opioid withdrawal symptoms. In Israel, 152 prisoners aged 20‐58 years old were included in a study, 69 male former users of heroin who received since their detention methadone and 83 non‐opioid users. The prevalence of sleep bruxism and awake bruxism was statistically significantly higher among the methadone users when compared to the control group. This outcome suggested a direct or indirect association between methadone maintenance therapy and sleep bruxism and awake bruxism. However, several potential confounders were involved, such as previous opioid abuse, previous or current alcohol abuse, anxiety, stress and psychotropic medication.^25^ A scientific review on the latter study reported that the study could have been much more relevant with for instance a stricter adoption of the current standard of reference diagnostic grading for bruxism and a better control for possible confounding factors.^26^

### 3.7 Venlafaxine

Venlafaxine is, just as duloxetine, a SNRI. A systematic literature review on the association of the use of psychotropic medications with the presence of sleep bruxism mentioned that venlafaxine was positively related to sleep bruxism with odds ratio 2.28 and 95% confidence interval 1.34‐3.86.^7^ Additionally, a recent article reported the case of a 69‐year‐old man with the diagnosis of major depressive disorder. He received 150 mg/day venlafaxine, 2 mg/day lorazepam and 100 mg/day quetiapine. Subsequently, he developed awake bruxism. The authors’ clinical impression was that the patient's awake bruxism was secondary to venlafaxine use.^27^

## 4 ADDICTIVE SUBSTANCES POTENTIALLY INDUCING BRUXISM

### 4.1 Alcohol

In 60 healthy female subjects with a mean age of 23.0 ± 1.9 years, nightly masseter muscle electromyography was substantially associated with the amount of alcohol intake. Although bruxism was not assessed directly, the results of this study suggest a positive association of alcohol intake with sleep bruxism.^28^ Results of a systematic literature review revealed from two case‐control studies a moderate association between alcohol intake and sleep bruxism with odds ratio 1.9 and 95% confidence interval 1.2‐2.8.^29^

### 4.2 Heroin

Bruxism as side effect of the misuse of the opioid heroin is well‐known among people who provide care to addicted people. However, this side effect is not well‐documented in the scientific literature. Several reports on the oral health of heroin users are available, but data on bruxism as such have not been presented. A systematic literature review and meta‐analysis of the association between poor oral health and substance abuse concluded that addictive substance abusers, among whom many heroin users, had statistically significantly more clinically assessed non‐carious tooth loss (tooth wear). Tooth wear combines attrition (due to bruxism), abrasion and erosion.^30^ A recent publication from Australia reports non‐specified bruxism among the oral adverse effects of illicit drug use, including heroin use.^31^

In conclusion: Use of heroin may induce non‐specified bruxism.

### 4.3 Methamphetamine

Methamphetamine, also known as *crystal meth*, *meth*, *tina* or *ice*, is related to amphetamine. Unlike amphetamine, methamphetamine is directly neurotoxic. Its euphoric and aphrodisiac qualities are stronger and longer lasting when compared to amphetamine. Methamphetamine was synthesised in Japan, and the highest prevalence of illegal use occurs in the United States, Oceania and parts of Asia. Meanwhile, it has become available in Europe, predominantly in Germany and the Czech Republic, but also in the United Kingdom and the Netherlands.^32^, ^33^

In the United States, a group of 301 adults of 18 years or older, who were dependent on methamphetamine, reported non‐specified bruxism in 22.3% of cases.^34^ In Germany, even 68% of a group of chronic methamphetamine abusers showed symptoms of non‐specified bruxism. When compared to a group of 100 control persons, statistically significantly more methamphetamine abusers had objectively assessed clinical symptoms of non‐specified bruxism, 39% versus 81%. The objectively assessed clinical symptoms were tooth attrition, dentin exposure and visible enamel cracks.^35^ A recent publication from Australia reports non‐specified bruxism among the oral adverse effects of illicit drug use, including methamphetamine use.^31^

### 4.4 Methylenedioxymethamphetamine

Just like methamphetamine, 3,4‐methylenedioxymethamphetamine (MDMA) is related to amphetamine and having stronger and longer lasting euphoric and aphrodisiac qualities than amphetamine. MDMA is also known as *ecstasy* and *xtc*, since MDMA is the active substance of ecstasy. A remarkable experiment was established in four male adult baboons. MDMA was administered via an intra‐gastric catheter. Behavioural observations revealed increased frequency of non‐specified bruxism as the dose of MDMA was increased.^36^

A theory suggests that MDMA induces the release of serotonin, which may result in reduced dopaminergic activity at the prefrontal cortex. Deficiency of dopamine contributes to the development of bruxism. In addition, MDMA‐induced release of serotonin and norepinephrines may overstimulate the trigeminal motor neurons which control the position and movements of the mandible and the reflexes of the masseter muscles.^37^

### 4.5 Nicotine

From a systematic review of the scientific literature could be concluded that in two case‐control studies, smoking was moderately positively associated with sleep bruxism. Result of the statistical analysis was odds ratio 2.8 and 95% confidence interval 2.2‐3.5.^29^

A study in Finland among young twin adults found that self‐reported weekly bruxers were more than two times more likely to report heavy smoking than never bruxers, with odds ratio 2.5 and 95% confidence interval 1.8‐3.4.^38^ A subsequent study among twins with a mean age of 44 years elucidated that both monthly and rarely reported non‐specified bruxism was associated with both current and former cigarette smoking. Monthly reported non‐specified bruxism and current cigarette smoking: odds ratio 1.74 and 95% confidence interval 1.37‐2.22. Rarely reported non‐specified bruxism and current cigarette smoking: odds ratio 1.64 and 95% confidence interval 1.44‐1.86. Monthly reported non‐specified bruxism and former cigarette smoking: odds ratio 1.64 and 95% confidence interval 1.27‐2.11. Rarely reported non‐specified bruxism and former cigarette smoking: odds ratio 1.47 and 95% confidence interval 1.29‐1.67. Weekly non‐specified bruxism was associated with current smoking: odds ratio 2.85 and 95% confidence interval 2.26‐3.61. Current smokers smoking 20 or more cigarettes a day reported weekly non‐specified bruxism 1.61 to 1.97 more likely than those smoking less: 95% confidence interval 1.02‐2.54 and 1.16‐3.37, respectively.^39^

In conclusion: Use of nicotine, by smoking cigarettes, may induce non‐specified bruxism.

### 4.6 Piperazines

Piperazines are a broad class of synthetic chemical compounds. The combination of 1‐(3‐trifluoromethylphenyl)piperazine (TFMPP) and 1‐benzylpiperazine (BZP) is often used as recreational drug because of its psychoactive properties. In the scientific literature, one article describes three young patients who presented on one evening, independently of each other, at an emergency department. All had dissociative‐type symptoms, nausea and signs consistent with sympathomimetic toxicity, including awake bruxism in two of them. All had ingested four tablets, thought to be MDMA, purchased in the same club venue. Serum analysis demonstrated the presence of TFMPP and BZP, not of MDMA. They improved with conservative management and observation within twelve hours.^40^

## 5 MEDICAMENTS POTENTIALLY ATTENUATING BRUXISM

### 5.1 Amitriptyline

Amitriptyline is a tricyclic antidepressant, used to treat several mental diseases. Using a double‐blind and randomised experimental design, ten adult subjects with sleep bruxism were administered amitriptyline and placebo, each compound over a period of one week. Neither the intensities and locations of pains nor the nocturnal masseteric electromyographic activities were significantly affected by the tricyclic antidepressant.^41^ These results were confirmed by a similar study.^42^ However, a case report on a 44‐year‐old woman presented an opposite finding. This woman, diagnosed with fibromyalgia, was prescribed duloxetine 60 mg/day. A few days later, her husband noted severe sleep bruxism. Duloxetine dosage was then reduced to 30 mg/day. As bruxism continued with this dosage, the therapy was discontinued with cessation of symptoms. Three weeks later, duloxetine therapy was restarted at the dosage of 60 mg/day. On the third day of treatment, sleep bruxism started again and amitriptyline therapy at the dosage of 10 mg/day was added to duloxetine therapy. The dosage of amitriptyline was incrementally adjusted to 25 mg/day. On the fourth day of combined therapy, sleep bruxism symptoms improved. Two months later, sleep bruxism symptoms were resolved.^43^

The results of two double‐blind and randomised research projects on the one hand and one case report on the other hand are contradictory. Scientifically, it could be concluded that the level of evidence of the double‐blind and randomised research projects is more valuable when compared to the level of evidence of one case report. However, taking the case report seriously, the conclusion could be that clinicians should be aware that amitriptyline may be a proper treatment for duloxetine‐induced sleep bruxism.

When considering prescribing amitriptyline, attention should be paid to common side or adverse effects as reported by the US Food and Drug Administration (FDA), such as dizziness, drowsiness, headache, constipation, weight gain, xerostomia and hyposalivation.

### 5.2 Botulinum toxin A

Botulinum toxin A is a neurotoxin protein which can produce a blockage in the linkage of acetylcholine towards muscle endplates. Injected intra‐muscularly in a therapeutic dosage, it produces muscle relaxation. It is used to control non‐specified bruxism in patients with myofascial pain of temporomandibular muscles. A systematic literature review, which included four studies, reported the efficacy of this treatment for patients with sleep bruxism. Two of these studies diagnosed sleep bruxism using polysomnography/electromyography, whilst the other two based the diagnosis on history taking and clinical examination.^44^ However, the literature is still unclear about the dosage of botulinum toxin A needed. An additional systematic literature review concluded that botulinum toxin A is a safe treatment for patients with sleep bruxism and awake bruxism, which offers superior efficacy when compared to control groups treated with placebo or traditional methods. Four randomised controlled trials met the inclusion criteria. The diagnostic methods used were polysomnography, assessment of occlusal forces, objective measurements of mandibular movements, questionnaires and visual analogue scales.^45^

As an important notice, adverse effects of bone loss at the condylar and alveolar regions of the mandible have been demonstrated after treatment with botulinum toxin A. Presumably, the bone loss develops due to localised paresis of the muscles, inducing mineral density. This phenomenon is called disuse osteopenia.^46^

Research of larger samples and over a long time period is needed to further establish both the efficacy and the safety of botulinum toxin A in the treatment of bruxism.

When considering prescribing botulinum toxin A, attention should be paid to common side or adverse effects as reported by the FDA, such as asthenia, blepharoptosis, dysphagia, myasthenia, neck pain, visual disturbance, xerostomia and hyposalivation.

### 5.3 Buspirone

Buspirone is an anxiolytic. In twenty case reports, non‐specified bruxism induced by several antidepressants has been found to improve by additional medication with buspirone.^19^ However, other case reports have demonstrated that non‐specified bruxism can also be reduced or terminated by dosage reduction of the medications.^47^ Together, these data suggest that the addition of 5‐10 mg up to 3 times daily may be an effective option for alleviating antidepressant‐associated non‐specified bruxism, particularly in patients who may not tolerate dose reduction or medication cessation.

A case was reported on a 7‐year‐old boy, previously diagnosed with pervasive developmental disorder‐not otherwise specified (PDD‐NOS) and moderate mental retardation, showing not‐medication‐induced non‐specified bruxism. Treatment with buspirone was started in a gradually increasing dosage. Once on 5 mg three times daily, the boy's parents noted that his bruxism had stopped throughout the day and night. At follow‐up 9 months after buspirone initiation, the non‐specified bruxism remained improved, but afternoon bruxism had recurred. At this time, buspirone was increased to 7.5 mg three times a day, resulting within weeks in the cessation of bruxism. Due to continued daytime sedation and night‐time awakenings, an attempt was made to reduce the buspirone dose to 5 mg thrice daily. This reduction resulted in a return of non‐specified bruxism and resultant dose increase back to the most effective dose of 7.5 mg thrice a day.^48^

A case report, not mentioned in the review by Garrett and Hawley,^19^ reported atomoxetine‐induced awake bruxism and sleep bruxism. Buspirone was added to the medication with atomoxetine at an initial dosage of 5 mg/day and gradually increased to 10 mg/day. Within approximately 10 days, the awake bruxism and the sleep bruxism improved significantly.^22^

The 6‐year‐old girl, who presented with sleep bruxism after fluoxetine treatment (see paragraph 2.3), was treated successfully with buspirone, which was confirmed with clear on‐off‐on treatment sequence.^20^

The mother of a 7‐year‐old boy, diagnosed with ADHD, reported that the boy showed bruxism in various phases of sleep every night during about two hours. Buspirone 5 mg/day was started, and after one week, sleep bruxism declined significantly. By the end of six‐week medication without sleep bruxism, the mother decided to withdraw the medication. One week later, sleep bruxism reappeared.^49^

In conclusion: Buspirone seems a medication which can be used to attenuate medication‐induced and not‐medication‐induced sleep bruxism and awake bruxism effectively.

When considering prescribing buspirone, attention should be paid to common side or adverse effects as reported by the FDA, such as dizziness, headache, nausea, nervousness, lightheadedness and excitement.

### 5.4 Clonazepam

Clonazepam is a benzodiazepine with anticonvulsant properties. A group of 21 middle‐aged people suffering from sleep bruxism received 1 mg/day clonazepam. When compared to placebo, clonazepam yielded polysomnographically a statistically significant reduction of sleep bruxism.^50^

Safety procedures are necessary with benzodiazepines. They are relatively safe for two to four weeks use, but their safety has not been established beyond that period. Dependence develops in approximately 50% of patients who use benzodiazepines for longer than one month.^51^ Other common side or adverse effects as reported by the FDA are drowsiness, dizziness, ataxia, depression and somnolence.

### 5.5 Clonidine

Clonidine is an antihypertensive, also used to treat ADHD, migraine, chronic pain and psychiatric disorders. Using polysomnographic sleep laboratory recordings, it was demonstrated that clonidine, when compared to placebo, reduced sleep bruxism statistically significantly.^52^ Several years later, a Japanese study confirmed this finding.^53^

In paragraph 2.2, the case report of a 9‐year‐old girl with ADHD is described. She received 18 mg/day methylphenidate which induced sleep bruxism. Two days after treatment with clonidine, the sleep bruxism abated entirely and did not reappear.^17^

When considering prescribing clonidine, attention should be paid to common side or adverse effects as reported by the FDA, such as drowsiness, dizziness, fatigue, nausea, constipation, xerophthalmia, xerostomia and hyposalivation.

### 5.6 Clozapine

The atypical antipsychotic clozapine is mainly used for schizophrenia that does not improve following the use of other antipsychotic medications and for psychiatric problems in Parkinson's disease. A 56‐year‐old man was suffering from schizophrenia with frequent relapse and remission for the last fourteen years. At the end of eight years of antipsychotic therapy, he developed awake bruxism. Since he was in remission, he was not given any antipsychotic drugs for the next six years, but he continued to exhibit awake bruxism. Subsequently, he developed paranoid symptoms and was started on a regimen of clozapine 25 mg/day. This was increased to 150 mg/day over a period of six weeks. With clozapine, his paranoid symptoms and awake bruxism were controlled. At the end of sixteen weeks of treatment with clozapine, the awake bruxism disappeared, and he was stabilised on the same dose of clozapine without recurrence of awake bruxism.^54^

When considering prescribing clozapine, attention should be paid to common side or adverse effects as reported by the FDA, such as weight gain, tremor, dizziness, drowsiness, lightheadedness, blurred vision, tachycardia, constipation, xerostomia, hyposalivation and sialorrhoea.

### 5.7 Gabapentin

Besides the benzodiazepine with anticonvulsant properties clonazepam, also the benzodiazepine with anticonvulsant properties gabapentin appeared to have the capability to attenuate bruxism. Using polysomnographic recordings, ten persons experiencing not‐medication‐induced sleep bruxism showed statistically significant reductions in several sleep bruxism symptoms.^55^ A 43‐year‐old depressive woman used the SSRI fluoxetine 20 mg/day during the previous four months. During this whole time period, she had experienced sleep bruxism symptoms. Gabapentin 150 mg/day was initiated additionally to fluoxetine and titrated up to 300 mg/day. Beginning from the fifth day, the symptoms of sleep bruxism disappeared and did not reappear during one‐year follow‐up.^52^

In conclusion: Gabapentin seems a medication which can be used to attenuate medication‐induced and not‐medication‐induced sleep bruxism effectively.

When considering prescribing gabapentin, attention should be paid to common side or adverse effects as reported by the FDA, such as ataxia, dizziness, drowsiness, fatigue, nausea, vomiting and tremor.

### 5.8 Hydroxyzine

Hydroxyzine is an antihistamine used for treatment of anxiety. The parents of three children, who used to co‐sleep with their parents, reported not‐medication‐induced sleep bruxism in their children. Hydroxyzine 10 to 25 mg/night was administered, and after taking it for one month, the parents reported a significant reduction of sleep bruxism severity according to their scores on a visual analogue scale.^56^ A randomised, double‐blind, placebo‐controlled clinical trial among children showed through the outcome of a visual analogue scale test that hydroxyzine statistically significantly decreased sleep bruxism severity more than placebo.^57^

In conclusion: Hydroxyzine seems a medication which can be used to attenuate not‐medication‐induced sleep bruxism effectively.

When considering prescribing hydroxyzine, attention should be paid to common side or adverse effects as reported by the FDA, such as fatigue, sleepiness, disturbed coordination and stomach distress.

### 5.9 Levodopa and dopamine agonists

Levodopa is precursor to the neurotransmitter dopamine. It is applied as a medication to reduce the motor symptoms of Parkinson's disease.^58^ Using polysomnographic sleep laboratory recordings, it was demonstrated that the medication levodopa (_L_‐dopa) exerts an attenuating effect on sleep bruxism.^59^ Another study found that administration of the dopamine agonist bromocriptine reduced sleep bruxism, supporting previous suggestions that the central dopaminergic system may be involved in the modulation of sleep bruxism.^60^ However, a randomised, double‐blind, placebo‐controlled study demonstrated that a nightly dose of bromocriptine did not exacerbate or reduce sleep bruxism.^61^ Additionally, a research group in Sweden found that the dopaminergic agonist pramipexole had no attenuating effect on sleep bruxism.^62^

Conclusion: More scientific studies are needed to discover evidence for the effect of dopamine agonists on sleep bruxism.

When considering prescribing levodopa or a dopamine agonist, attention should be paid to common side or adverse effects as reported by the FDA, such as dizziness, lightheadedness, nausea, vomiting, loss of appetite, insomnia and headache.

### 5.10 Propranolol

Propranolol is a medication of the beta blocker class, used to treat among others hypertension, arrythmia and migraine headache. A person with heavy sleep bruxism was treated by propranolol. His nocturnal masseter muscle activity, registered bilaterally with EMG recordings, decreased by 72%.^63^ However, in a study among 25 persons with sleep bruxism propranolol did not reduce polysomnographically recorded sleep bruxism.^52^

When considering prescribing propranolol, attention should be paid to common side or adverse effects as reported by the FDA, such as bradycardia, diarrhoea, nausea, fatigue, hair loss and xerophthalmia.

### 5.11 Quetiapine

The antipsychotic quetiapine is used for the treatment of schizophrenia, bipolar disorder and major depressive disorder. An article described five patients who were treated with SSRIs and presented with non‐specified bruxism. Between 25 and 50 mg/day of quetiapine was sufficient to cease the non‐specified bruxism after a few days.^64^

When considering prescribing quetiapine, attention should be paid to common side or adverse effects as reported by the FDA, such as dizziness, constipation, nausea, vomiting, weight gain and increased appetite.

### 5.12 Trazodone

Trazodone is a serotonin antagonist and reuptake inhibitor (SARI), mainly used as antidepressant. A 34‐year‐old man with a history of bipolar disorder was treated with carbamazepine, clonazepam and escitalopram. He complained of sleep bruxism. The SSRI escitalopram was suspected to cause the sleep bruxism. Trazodone was added gradually before sleep until the target dose of 200 mg/day. The man and his wife reported marked attenuation of the sleep bruxism.^65^

When considering prescribing trazodone, attention should be paid to common side or adverse effects as reported by the FDA, such as dizziness, headache, blurred vision, lightheadedness, nausea, nervousness, decreased sexual desire, xerostomia and hyposalivation.

## 6 DISCUSSION

The objective of this narrative literature review was to provide information on classes of medications, individual medications and addictive substances potentially inducing or aggravating sleep bruxism and/or awake bruxism and on medications potentially attenuating sleep bruxism and/or awake bruxism. Summarising, Tables 1, 2 and 3 list statements of, respectively, classes of medications, individual medications and addictive substances which potentially induce or aggravate sleep bruxism and/or awake bruxism, whereas Table 4 lists the medications potentially attenuating sleep bruxism and/or awake bruxism.

**TABLE 1:** List of classes of medications, potentially inducing or aggravating sleep bruxism and/or awake bruxism

Classes of medications	Type of bruxism	Subclasses of medications	Individual medications
Anticonvulsants	Non‐specified	Aldehydes	Paraldehyde
		Aromatic allylic alcohols	Stiripentol
		Barbiturates	Barbexaclone
			Methylphenobarbital
			Phenobarbital
		Benzodiazepines	Diazepam
			Lorazepam
			Midazolam
		Carbamates	Felbamate
		Carboxamides	Carbamazepine
			Eslicarbazepine acetate
			Oxcarbazepine
		Fatty acids	Divalproex sodium/Valproate semisodium
			Progabide
			Sodium valproate
			Tiagabine
			Valproic acid/Valproate
			Vigabatrin
		Fructose derivates	Topiramate
		Hydantoins	Ethotoin
			Fosphenytoin
			Mephenytoin
			Phenytoin
		Oxazolidinediones	Ethadione
			Paramethadione
			Trimethadione
		Pyrimidinediones	Primidone
		Pyrrolidines	Brivaracetam
			Etiracetam
			Levetiracetam
		Succinimides	Ethosuximide
			Mesuximide
			Phensuximide
		Sulphonamides	Acetazolamide
			Sultiame
			Zonisamide
		Triazines	Lamotrigine
		Ureas	Phenacemide
			Pheneturide
		Valproylamides	Valnoctamide
			Valpromide
		Others	Perampanel
			Pyridoxine
Phenethylamines	Sleep and awake		Amphetamine
			Dextroamphetamine
			Levoamphetamine
			Lisdexamfetamine
			Methylphenidate
Selective serotonin reuptake inhibitors	Sleep and awake		Citalopram
			Dapoxetine
			Escitalopram
			Fluoxetine
			Fluvoxamine
			Paroxetine
			Sertraline
			Vilazodone

**TABLE 2:** List of individual medications, potentially inducing or aggravating sleep bruxism and/or awake bruxism

Individual medication	Class of medication	Type of bruxism
Aripiprazole	Atypical antipsychotics	Non‐specified
Atomoxetine	Norepinephrine reuptake inhibitors	Sleep and awake
Duloxetine	Serotonin‐norepinephrine reuptake inhibitors	Sleep
Flecainide	Antiarrhythmics	Awake
Ketotifen	Selective antihistamines	Sleep
Methadone	Opioids	Sleep and awake
Venlafaxine	Serotonin‐norepinephrine reuptake inhibitors	Sleep and awake

**TABLE 3:** List of addictive substances, potentially inducing or aggravating sleep bruxism and/or awake bruxism

Addictive substances	Type of bruxism
Alcohol	Sleep
Heroin	Non‐specified
Methamphetamine	Non‐specified
Methylenedioxymethamphetamine	Non‐specified
Nicotine	Non‐specified
Piperazines	Awake

**TABLE 4:** List of medications with strong, moderate or questionable indications for capability to attenuate sleep bruxism and/or awake bruxism

Medicaments	Type of bruxism	Strong	Moderate	Questionable
Amitriptyline	Sleep			X
Botulinum toxin A^a^	Sleep and awake	X		
Buspirone	Sleep and awake	X		
Clonazepam^b^	Sleep		X	
Clonidine	Sleep		X	
Clozapine	Awake			X
Gabapentin	Sleep	X		
Hydroxyzine	Sleep	X		
Levodopa/dopamine agonists	Sleep		X	
Propranolol	Sleep			X
Quetiapine	Non‐specified		X	
Trazodone	Sleep			X

^a^ Its safety is questionable.

^b^ Its safety is questionable beyond a two to four weeks period.

In order to meet the objective of this study, it was decided to establish a narrative literature review, summarising primary studies and case reports. The narrative literature review identified several gaps in the current knowledge and generated some hypotheses from existing knowledge, which are considered useful results of a narrative literature review.^66^ For several subparagraphs, a conclusion or suggestion could be provided. Generally, there are insufficient evidence‐based data to draw definite conclusions concerning medications and addictive substances inducing or aggravating sleep bruxism and/or awake bruxism as well as concerning medications attenuating sleep bruxism and/or awake bruxism. The literature is controversial and based mostly on anecdotal case reports. More controlled, evidence‐based research on these under‐explored topics is needed. Future research on medications potentially attenuating sleep and/or awake bruxism may focus at first on medications with strong indications for capability to attenuate bruxism as mentioned in Table 4: botulinum toxin A, buspirone, gabapentin and hydroxyzine.

With regard to the information provided on medications potentially attenuating sleep bruxism and/or awake bruxism, it should be stressed that bruxism only requires management in case of severe negative health outcomes, such as severe mechanical tooth wear (attrition) and high‐intensity pain of masticatory muscles and/or temporomandibular joints.^1^ In other cases, bruxism should be left untreated because of the condition's suggested positive health outcomes, such as improving upper airway patency during sleep which contributes to preventing apnoea‐hypopnoea events in individuals with obstructive sleep apnoea.^67^ In case management of bruxism is deemed necessary, it is recommended to follow the results of systematic literature reviews.^10^, ^68^

## 7 CONCLUSION

There are insufficient evidence‐based data to draw definite conclusions concerning medications and addictive substances inducing or aggravating sleep bruxism and/or awake bruxism as well as concerning medications attenuating sleep bruxism and/or awake bruxism.

## CONFLICT OF INTEREST

The authors of this manuscript declare that they have no conflict of interest. They did not receive any funding for this study.

## AUTHORS’ CONTRIBUTIONS

Cees de Baat, Merel Verhoeff and Frank Lobbezoo conceptualised and prepared the literature review. Cees de Baat performed the literature review, and Merel Verhoeff, Jari Ahlberg, Daniele Manfredini, Ephraim Winocur and Frank Lobbezoo contributed to it. Cees de Baat, Petra Zweers, Fred Rozema and Arjan Vissink analysed and interpreted the data of the selected articles. The original draft was written by Cees de Baat. Merel Verhoeff as well as Petra Zweers and Frank Lobbezoo performed a first editing. A second editing was performed by Jari Ahlberg, Daniele Manfredini, Ephraim Winocur, Fred Rozema and Arjan Vissink. All authors gave their final approval and agreed to be accountable for all aspects of the publication.

### Peer Review

The peer review history for this article is available at https://publons.com/publon/10.1111/joor.13061.
